# Symbolic and Graphical Representation Scheme for Sensors Deployed in Large-Scale Structures

**DOI:** 10.3390/s130809774

**Published:** 2013-07-31

**Authors:** Hyo Seon Park, Yunah Shin, Se Woon Choi, Yousok Kim

**Affiliations:** 1 Department of Architectural Engineering, Yonsei University, 134 Shinchon-dong, Seoul 110-732, Korea; E-Mails: hspark@yonsei.ac.kr (H.S.P.); yunah@yonsei.ac.kr (Y.S.); 2 Center for Structural Health Care Technology in Buildings, Yonsei University, 134 Shinchon-dong, Seoul 110-732, Korea; E-Mail: watercloud@yonsei.ac.kr

**Keywords:** structural health monitoring, large-scale structures, wireless sensor network, sensor network management

## Abstract

As wireless sensor network (WSN)-based structural health monitoring (SHM) systems are increasingly being employed in civil infrastructures and building structures, the management of large numbers of sensing devices and the large amount of data acquired from WSNs will become increasingly difficult unless systematic expressions of the sensor network are provided. This study introduces a practical WSN for SHM that consists of sensors, wireless sensor nodes, repeater nodes, master nodes, and monitoring servers. This study also proposes a symbolic and graphical representation scheme (SGRS) for this system, in which the communication relationships and respective location information of the distributed sensing components are expressed in a concise manner. The SGRS was applied to the proposed WSN, which is employed in an actual large-scale irregular structure in which three types of sensors (75 vibrating wire strain gauges, 10 inclinometers, and three laser displacement sensors) and customized wireless sensor nodes are installed. The application results demonstrate that prompt identification of sensing units and effective management of the distributed sensor network can be realized from the SGRS. The results also demonstrate the superiority of the SGRS over conventional expression methods in which a box diagram or tree diagram representing the ID of sensors and data loggers is used.

## 1. Introduction

Structural health monitoring (SHM) has been attracting increasing interest as a method to determine the structural responses and evaluate the safety of civil infrastructure and building structures based on sensor technology [1–7]. Specifically, the characteristics of super-tall or large-scale structures require considerable caution in terms of the safety and accuracy of the construction process [8–12]. Conventional wire-based SHM has been shown to have certain limitations in applications involving actual structures, including the use of long and complex cable connections between sensors and data loggers, which is associated with high costs, not only in the initial installation, but also for maintenance during operation [13]. For these reasons, there has been considerable interest in wireless sensor networks (WSNs) because they represent a key technology for those who require effective SHM in large-scale structures [14,15]. The number of sensors used in SHM to obtain reliable data and evaluate structural safety is expected to increase drastically as the size and height of structures increases. Furthermore, as the critical issues (e.g., power efficiency, transmission limitations due to bandwidth restrictions and distance) involved in WSN applications are resolved, the development of a large-scale WSN topology in actual structures will become feasible.

As WSNs are actively employed, various wireless sensing units (sensor nodes) have been designed and developed based on the characteristics of measuring devices, such as strain gauges [16] and accelerometers [17]. These traditional measuring sensors are connected to wireless sensor nodes that mainly function to collect data from measuring devices, process these data, and transfer information via wireless communication protocols. The limited transmission distance of wireless sensor nodes requires the adoption of a multi-hop network topology for the transmission of measured data [17]; accordingly, WSNs become increasingly complex, and data measured from a certain point are transferred via various intermediate wireless sensing units until the data are finally delivered to the main server (*i.e.*, the final data repository). As a result, the management of devices comprising WSNs and the large amounts of data acquired from these devices will become increasingly difficult as the size of buildings and the extent of SHM increase [18].

The commonly used method to express sensor networks consisting of multiple sensors and different types of data loggers is to express the IDs of sensors and data loggers (nodes) as a combination of letters and numerals [19–21] or to express them symbolically in a coordinated form [22–24].

Recently, numerous low-cost and low-power sensors associated with WSNs have become densely distributed in surveillance areas. In the Guangzhou New TV Tower [25,26], in which approximately 600 sensors are used, the sensors are divided into four types, namely, portable sensors and fixed devices located in the inner structure, outer structure, and antenna mast, with the number of sensors for each type expressed accordingly. The communication relationship for each type of sensor is vertically graphed, making it easy to understand the overall system; however, the detailed notation corresponding to the various types of equipment remains unresolved, and the accurate surface locations and communication relationships between the different types of equipment cannot be comprehended.

A total of 432 sensors of 11 different types are installed in the Shanghai Tower [27], and these sensors are vertically expressed as the number of sensors of each sensor type as a function of the elevation of the building in a manner similar to the Guangzhou New TV Tower. In addition, the monitoring zone in the Shanghai Tower is divided vertically, and each zone is classified from the ground to the top by a series of numbers. However, it is difficult to comprehend the location of sensors on the architectural plan, communication relationships, and overall system composition. The name of each sensor is expressed in the vertical zone, leading to the requirement for more space than is shown in the diagram and thus more time to understand the overall composition.

In the SHM of a 31-story office building in Tokyo performed in 2007 [28], which was undertaken to consider the safety issues that arise during a typhoon, the location of attached equipment was included on the plan, although the location was expressed using structural drawings and was only applied to the sixth and 13th floors, making it difficult to comprehend the flow of the overall system.

As illustrated by the aforementioned cases, there is a noticeable lack of research on systematic expressions of the sensor networks in structures that are divided into various spaces and comprise multiple vertical layers. For example, in addition to the location of measuring devices, information related to each component (*i.e.*, sensors and wireless sensor nodes) and their associated relationships should be clearly indicated for efficient and prompt maintenance and management in the event that a device malfunction is detected or a dangerous response index is recorded. Furthermore, from the perspective of a manager with no experience in configuring a sensor network at such a site, there is a need to develop a new succinct expression method to provide knowledge of the system.

This study proposes a symbolic and graphical representation scheme (SGRS) for a WSN for the SHM of large-scale structures and a strategy for the effective maintenance and management of such structures. A horizontal and vertical representation scheme is used in a flexible manner to reflect the specificity of each site when applied to various types and scales of structures. The SGRS is applied to the long-term monitoring of the structural responses of an actual structure currently under construction to evaluate its practicality. A large-scale irregular structure of five stories (three underground) with a total floor area of 85,320 m^2^ was used to provide an example application of this SHM system. In the structure, the vertical deflection, tilt, and strain of the members at vulnerable points of stability were the main objects of monitoring. The SGRS was applied for 41 data loggers and a total of 88 sensors of three types, namely, 75 vibrating wire strain gauges (VWSGs), 10 inclinometers, and three laser displacement sensors. In the proposed SGRS, the information obtained from measuring devices is expressed together with the relationship among the employed devices in a compact manner, which enables the network administrator to efficiently manage the sensor networks and measured data.

## 2. The Practical SHM of Large-Scale Structures

**Figure 1.**
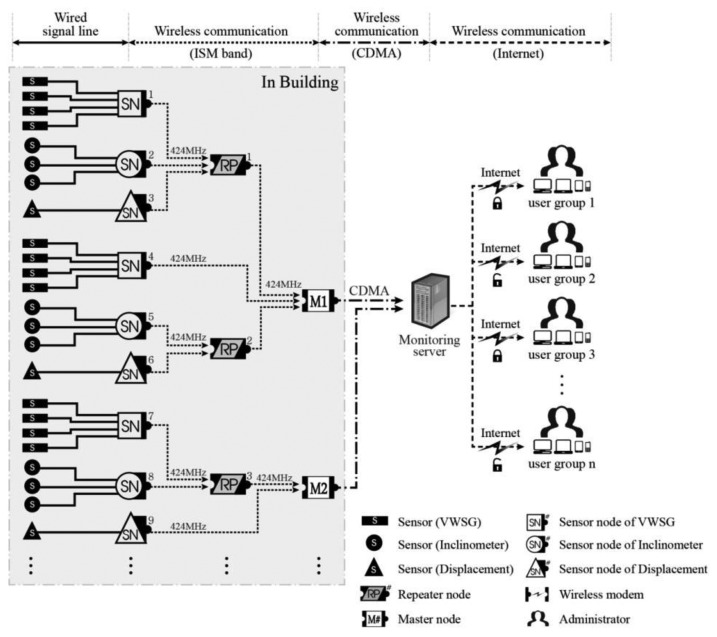
WSN for SHM.

Figure 1 presents the WSN for an SHM, composed of sensors (S), sensor nodes (SN), repeater nodes (RP), master nodes (MN), monitoring servers, and administrators. Sensors, which are measuring devices, do not have a function for wireless transmission and are thus connected by a signal line to the sensor node, which performs data processing and wireless transceiver functions.

The measured data in the sensor nodes must pass through the monitoring server to be transmitted to the wireless terminals held by several administrators. The wireless transceivers that perform this role are called the repeater node and master node. Considering the stability of wireless communication at an actual site with structural or nonstructural partitions hindering wireless communication, data transmission from the sensor node to the master node uses the 424 MHz Industrial Scientific and Medical (ISM) band, which has better diffractive characteristics than the existing 2.4 GHz communication standard. The repeater nodes in the intermediate process of this stage can also be used as an alternative option for wireless communication. These nodes perform a relay function between the sensor node and master node to solve transmission problems that can arise due to long communication distances or a complicated structural configuration that hinders direct data transmission due to obstructive elements. The master node can collect dozens of instances of sensor data; therefore, when located in an area in which wireless communication is possible, even a small number of master nodes can send and receive a large amount of sensor data simultaneously. This master node receives data from the sensor nodes and sends them to the monitoring server inside the central monitoring center located away from the structure using the code division multiple access (CDMA) method. CDMA communication is robust in terms of security and interference, and the coded signal of a specific user is perceived as noise by other users. Thus, there is no distance restriction, and there is only a low possibility of data noise occurring from long-distance transmissions. It is also possible to share limited resources with many users. For this reason, regardless of the distance from the monitoring site, the transmitted signal can be decoded and demodulated using wireless terminals, such as a PC, notebook, tablet, or mobile phone, such that administrators can check data at any time and from any location.

In this system, three types of sensors, namely, VWSGs, inclinometers, and laser displacement sensors, are used to measure structural responses, namely, the strain, angle of rotation, and deflection, respectively. In addition, the wireless sensor nodes corresponding to each measuring devices have been developed and are employed in this system to flexibly respond to the site specificity of various structure configurations.

## 3. A Symbolic and Graphical Representation Scheme of a WSN for SHM

The advantage of the SGRS proposed here is that it is possible to promptly comprehend the location of a sensor or data logger using 3D information instead of general vertical or horizontal 2D expressions. To establish the SGRS of the sensor network, the monitoring zone is configured to reflect the specificity of the structure for SHM, and its location information is used as the basic notation unit.

### 3.1. 3D Zoning Method (Graphical Representation)

The greatest advantage of the symbolic and graphical representation scheme is that when fixed monitoring zoning regulations are defined for each structure, the scheme is applicable to all structures, including those with complicated and varied forms. Areas that become objects for monitoring within a structure are typically structurally critical; therefore, the existing notation that uses a plan at the structural level or the original architectural drawing is rather inefficient when used in this manner for large-scale structures. As a result, a sequential categorization of the site zoning, vertical zoning, and horizontal zoning is performed.

#### 3.1.1. Site Zoning in Multisite Monitoring

**Figure 2.**
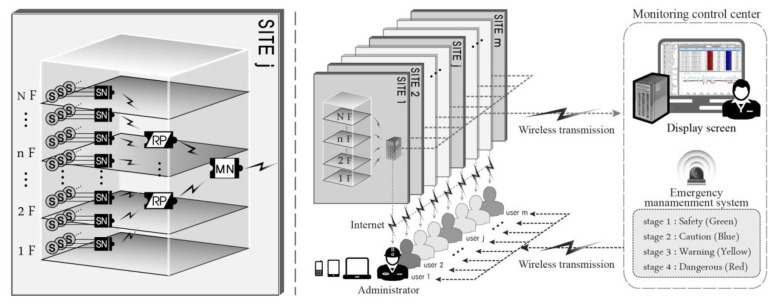
Site zoning of an SHM system (F: Floor).

The integrated operation and management of the multisite monitoring system for m sites is illustrated in Figure 2. The structural response information from each site is assembled into the final data collection device of the master node and then wirelessly transmitted to the monitoring server at the monitoring control center. The monitoring control center synthetically evaluates the conditions of all monitoring sites. An emergency management system can also be operated in which administrators are notified of emergency situations through the recognition of lost data that occurs for various unexpected reasons (e.g., communication problems, noise, and abnormal sensor installation) and through analyses of data that deviate from the predefined safety threshold (e.g., yield stress or drift ratio). A site manager can directly check the structural health of the site using wireless terminal equipment, such as a notebook, tablet PC, or mobile phone, and the overall analysis can be received from the monitoring control center, making it is possible to respond actively and promptly in real time.

#### 3.1.2. Vertical Zoning in a Site

A single site can be enlarged from the m sites where monitoring is occurring simultaneously, and detailed vertical zoning can be performed to monitor the conditions at the relevant site. Here, the critical structural members requiring monitoring measurements differ according to the form and site configuration of the structure. These members are conceptually categorized to have constant regularity to fit the site characteristics of the building structure. In other words, the zoning regulations for the monitoring unit do not involve expressions of the plan or elevations of the actual structure; instead, a virtual 3D space is formed to be developed based on the major members subjected to measurement and monitoring activities.

**Figure 3.**
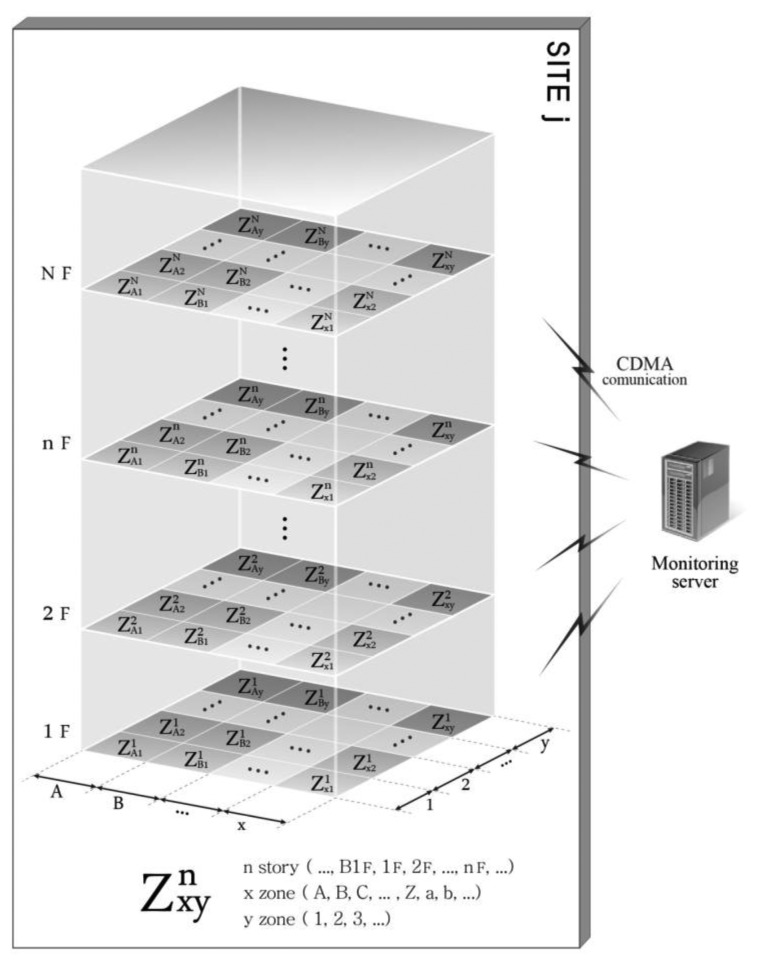
Vertical zoning in the site of an SHM system.

Figure 3 presents the vertical zoning of the monitoring situation of a random site j from the m monitoring sites. The site consists of *N* stories in total, and a virtual grid is formed according to the fixed rules of A1, A2, …, B1, B2, …, C1, … for each story to divide the monitoring area. In this case, the designations are not the x- and y-axes on a plan but instead signify a series of letters and Arabic numerals because there are buildings or structures that are irregular in shape and therefore cannot be divided into a regular grid on x- and y-axes. The XY zone (e.g., A1, A2, … B1, B2,…) of a random floor i signifies a monitoring unit, *i.e.*, the smallest unit that is monitored. This zone is expressed as ${\text{Z}}_{\text{XY}}^{\text{n}}$, where ${\text{Z}}_{\text{XY}}^{\text{n}}$ denotes the monitoring unit XY on the n^th^ floor. This expression indicates the location of the equipment and is used as a prefix for all notations.

#### 3.1.3. Horizontal Zoning in a Typical Story

The arrangement of wireless nodes on a single floor is determined according to the location of the sensors attached to the main members for the monitoring measurements, reflecting the condition of the floor. Here, all sensor and repeater nodes should be appropriately located within an area where wireless communication with the master node is possible.

**Figure 4.**
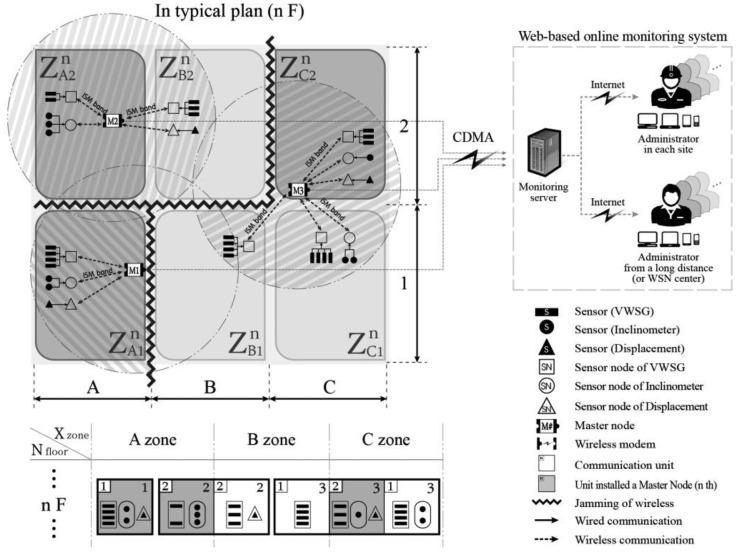
Horizontal zoning in a typical story of an SHM system.

Figure 4 presents the horizontal zoning of the monitoring configuration inside the typical plan of the nth floor from Figure 3. Regardless of the sensor type, a group of one or more sensor nodes located within a short distance, in which they can communicate with their final receiving master node in the communication flow, is called a monitoring unit. This monitoring unit is the smallest unit of the system, as depicted by the single closed quadrangle shown in Figure 4. All monitoring units are represented by a notation that expresses the location of the unit (e.g., the monitoring unit XY on the nth floor is ${\text{Z}}_{\text{XY}}^{\text{n}}$). The notation of monitoring units on a plan is identical to the rules explained in Figure 3. One or more monitoring units are restructured into a monitoring group. A single master node can be responsible for one or more monitoring units. Generally, due to the restrictions of wireless communication according to the actual site conditions, grouping can be organized in areas where wireless communication is possible. If wireless communication is performed through the exterior of the building due to open spaces, stairs, elevators, or windows, one master node can group monitoring units across several stories. In other words, one monitoring group refers to an area in which wireless communication is possible for a single master node. In the example of the n^th^ floor (Figure 4), a total of six monitoring units form three monitoring groups; the boundary of the monitoring groups is represented by the thick lines.

### 3.2. Expression Method (Symbolic Representation)

In the flow of communication connecting the sensors, sensor nodes, repeater nodes, and master nodes, the basic notation for each component is expressed by Marks 1 and 2 in Table 1, which illustrates the general rule for the SGRS. In front of all components, the notation for the location of the monitoring unit is specified, such as ${\text{Z}}_{\text{xy}}^{\text{n}}$ (the XY zone on the nth floor). Following the location, the type of component is expressed as S for sensor, SN for sensor node, RP for repeater node, and MN for master node. The final receiving master node number (δ) is written on the far right as a subscript for the components (e.g., *S_δ_*, *SN_δ_*, and *RN_δ_*). Additionally, the notations for sensors and sensor nodes can be added if different types of devices are employed in the WSN.

**Figure 5.**
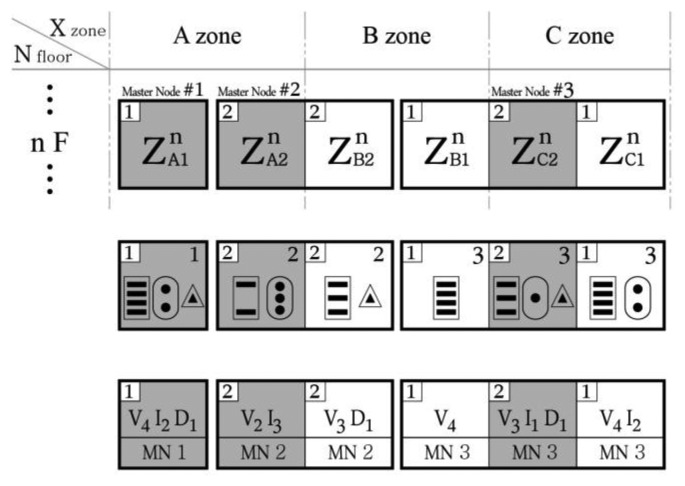
Method for conversion.

Figure 5 presents a simple depiction of the monitoring situation of the nth floor in Figure 4. First, it expresses ow the six monitoring units A1, A2, B1, B2, C1, and C2 on the nth floor are composed of three monitoring groups; the location notation of each unit is also given. Second, a graphic expression of the type of sensor and sensor node in each unit and how many sensors there are is presented. On the upper right of each unit, the number of the final receiving master node is indicated, thereby indicating the master node to which the relevant nodes belong. Third, a notational expression is also given to the unit, where V, I, and D correspond to VWSG, inclinometer, and laser displacement, respectively. The number written after each letter is the number of sensors of each type in the relevant unit. Below the sensor expression, the number of the final receiving master node is shown. In the monitoring group, the unit in which the master node is located is indicated by gray shading.

**Figure 6.**
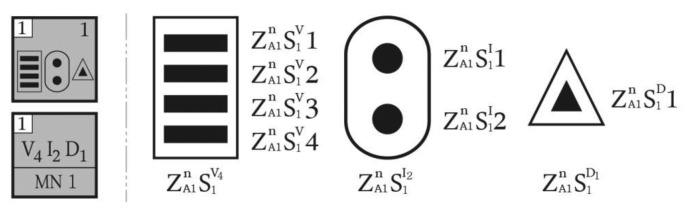
Notational system for each sensor of the A1 unit.

When the overall composition status is implemented at the level of the monitoring unit and monitoring group, the sensors and sensor nodes within each unit are numbered (Figure 5). Figure 6 presents the method used to number the components comprising unit A1 in Figure 4. The sensor node notation is as follows. The sensor node of VWSG becomes ${\text{Z}}_{\text{A}1}^{\text{n}}{\text{SN}}_{1}^{\text{V}4}$, the sensor node of the inclinometer becomes ${\text{Z}}_{\text{A}1}^{\text{n}}{\text{SN}}_{1}^{\text{I}2}$, and the laser displacement sensor node becomes ${\text{Z}}_{\text{A}1}^{\text{n}}{\text{SN}}_{1}^{\text{D}1}$. The notation of each sensor is as follows. The four VWSGs are ${\text{Z}}_{\text{A}1}^{\text{n}}{\text{S}}_{1}^{\text{V}}1$, ${\text{Z}}_{\text{A}1}^{\text{n}}{\text{S}}_{1}^{\text{V}}2$, ${\text{Z}}_{\text{A}1}^{\text{n}}{\text{S}}_{1}^{\text{V}}3$, and ${\text{Z}}_{\text{A}1}^{\text{n}}{\text{S}}_{1}^{\text{V}}4$; the two inclinometers are ${\text{Z}}_{\text{A}1}^{\text{n}}{\text{S}}_{1}^{\text{I}}1$ and ${\text{Z}}_{\text{A}1}^{\text{n}}{\text{S}}_{1}^{\text{I}}2$; and the one laser displacement sensor is expressed as ${\text{Z}}_{\text{A}1}^{\text{n}}{\text{S}}_{1}^{\text{D}}1$. Using this rule, it is possible to express all monitoring configurations. The location notation is written on the front of all notations such that the location of the equipment can be quickly identified. This format is also advantageous because it is easy to understand the type of sensor node, how many sensors are connected, and to which master node they belong.

## 4. Field Application

This section presents an example in which the convenience of management is increased by expressing the WSN for SHM in a real application to an irregular structure site (D-Building) under construction using the method proposed in Section 3.

### 4.1. Conventional Expression for the Network System

**Figure 7.**
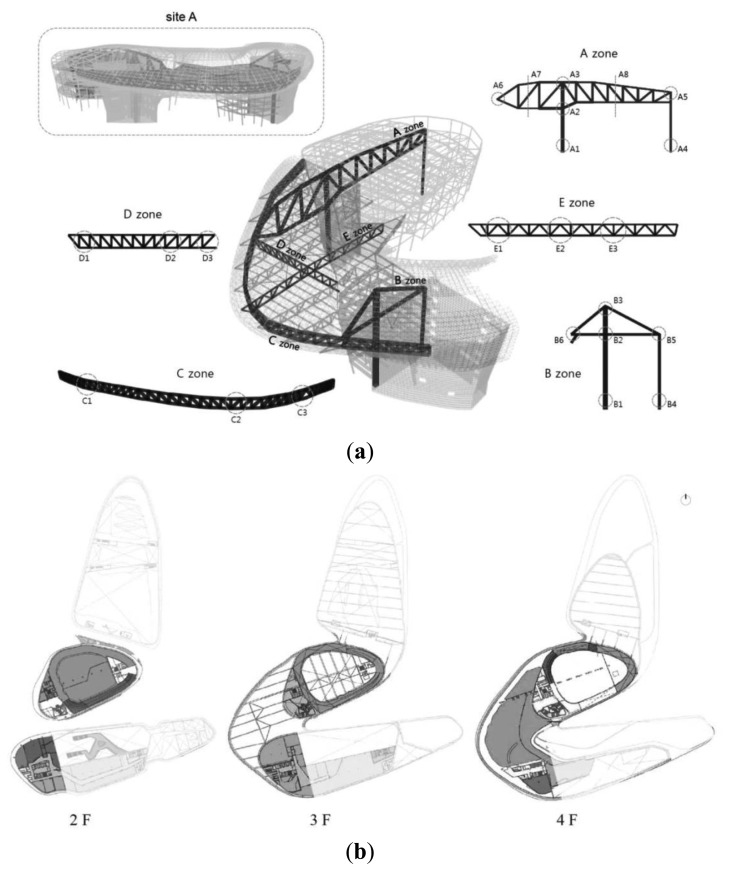
The irregular shaped buildings of D-Building. (**a**) Zoning of monitoring units; (**b**) The architectural floor plan.

D-Building is a large-scale irregular structure with three underground stories and four aboveground stories with a total floor area of 85,320 m^2^ that is currently under construction on a 61,585 m^2^ site. Figure 7a presents the main object members to be monitored at D-Building and the monitoring unit for each member. There are difficulties related to construction, such as exposed concrete and exterior panels with various free curvatures, complicated forms of space frames supporting the panels, irregular internal mega-spaces, and different uses of mega-members. Therefore, there is significant need for SHM to minimize the horizontal-vertical displacement and distortion error that can occur during the construction process. In addition, the plan is significantly different for each story, and the building itself is very large. Thus, there is considerable difficulty in terms of management and maintenance, as a prompt understanding of the monitoring status is not always possible. Figure 7b illustrates the WSN on the floor plan, which is generally used to illustrate the composition of the SHM system at D-Building. However, the organization of the system cannot be clearly viewed, and the compositional relationships between the equipment are unknown. Thus, it is difficult to indicate this information drawn to scale on the floor plan. In such a case, it is also necessary to have partially enlarged diagrams for each monitoring unit.

**Figure 8.**
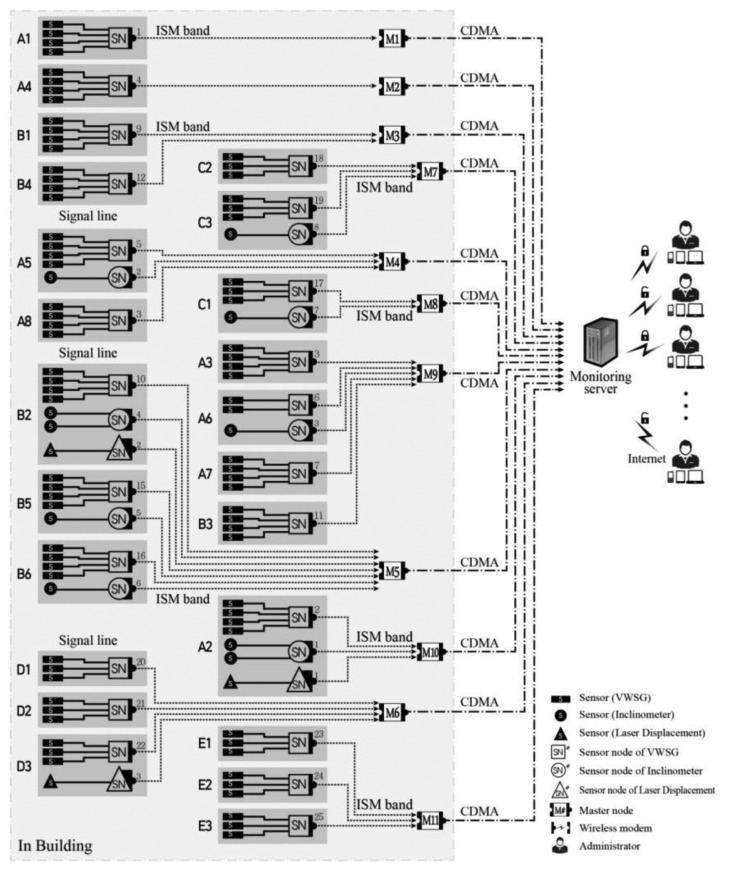
General designation method for communication at D-Building.

Figure 8 presents the composition of the WSN for SHM applied to D-Building and the communication flow chart. Generally, WSNs can be expressed in tree diagram form, as shown in Figure 8. The overall composition of the system can be understood at a glance using such a diagram. However, the notation becomes quite inconvenient and complicated as the number of components increases in the network, and such information as the location of each component and distinct numbering is omitted, thereby introducing a considerable disadvantage in terms of management efficiency. In addition, a considerable amount of time may be needed for managers other than the drafter to clearly understand the flow of any relevant system.

### 4.2. Application of the Proposed Method (SGRS) to D-Building

**Figure 9.**
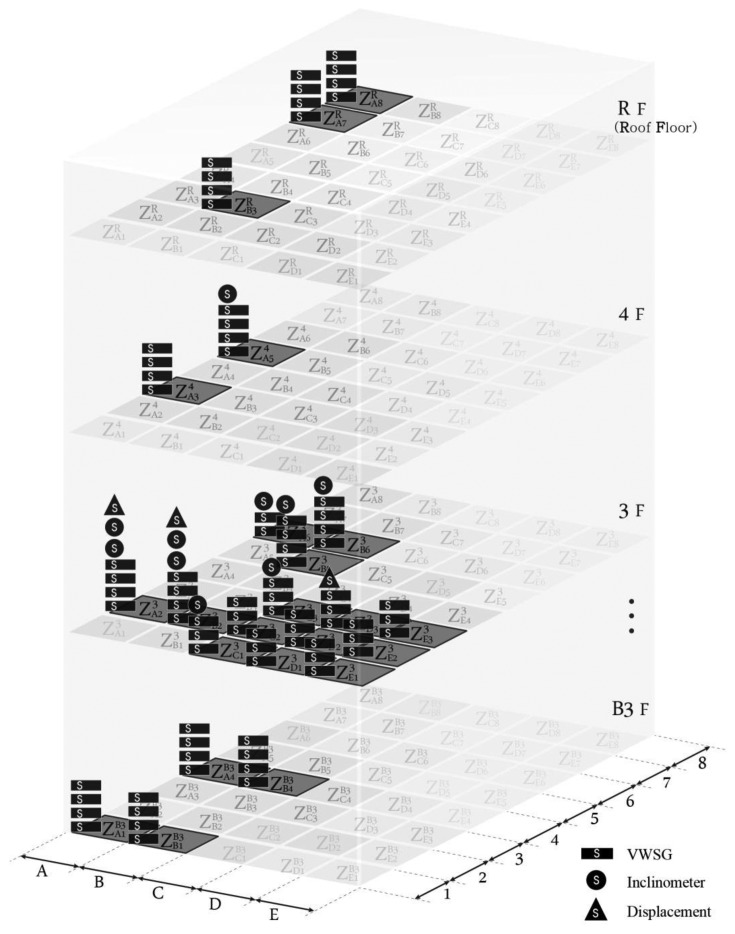
Conceptual 3D zoning diagram for D-Building.

Figure 9 presents the vertical zoning of the system for D-Building applied according to the scheme shown in Figure 3. The virtual zone is composed of each plan to conceptually express the monitoring unit in three dimensions. This image is provided to increase the understanding of the system, although it is not necessary when using the actual notation method.

As shown in Figure 7, D-Building is a highly irregular building that consists of mega-structural members, such as mega-columns, mega-trusses, edge trusses, and roof trusses. Therefore, it is almost impossible to divide the monitoring zones in a regular manner both in the horizontal and vertical directions. Instead, the large structural members of D-Building are categorized as A, B, C, D, and E with three to eight serial numbers assigned to each member.

**Figure 10.**
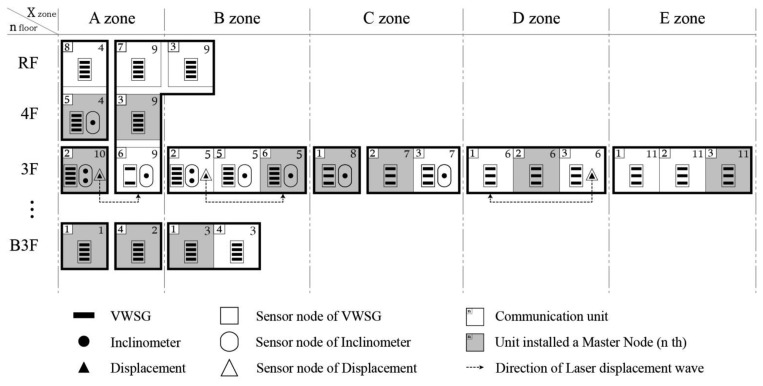
Graphical diagram expression for D-Building.

**Figure 11.**
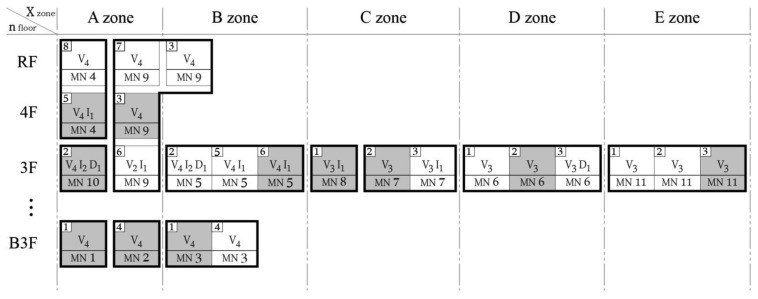
Simple notation for D-Building.

Two main approaches are used to express the composition of the WSN for SHM at D-Building. The first method uses the graphic expression introduced in Figure 10, and the second method uses the symbolic expression approach introduced in Figure 11.

Compared with Figure 8, Figure 10 summarizes a large amount of information regarding the system within a relatively small space. This graphical expression enables a clearer understanding of the distribution status of the wireless nodes and their communication relationships. Furthermore, both the location of the sensing equipment and the target measuring point are provided because the location of the sensor and target point varies depending on the sensor type. In the case of a laser displacement sensor, it is a noncontact type sensor and therefore located far from the measuring point. For this reason, this expression method indicates both units in which laser displacement sensors and target measuring points are located. That is, it first indicates a marking as the laser displacement sensor on the installed unit and uses dotted line arrows to indicate the target object unit (measuring point) in which the reflecting plate for the laser beam is located.

This approach is advantageous in that the characteristics of the sensors can also be considered and both the sensing device and target element can be monitored and managed simultaneously. Figure 11 presents a method using symbols to express the numbering assigned to each piece of equipment on a diagram of an identical size to that in Figure 10. The status and numbering of the sensor nodes shown in Figure 11 are identical to those aspects in Table 2.

## 5. Conclusions

This study proposes an SGRS to express the complex WSN that is employed in the monitoring of structural responses. The SGRS developed here symbolically expresses the locations and communication relationships of sensors and wireless nodes for each sensor and graphically expresses the composition and relationships pertaining to each sensor type, allowing the entire system to be represented in a concise and effective diagram. Through this scheme, the integrated management of monitoring systems is enabled even for super-tall buildings, large-scale structures, and multiple construction sites with different overall conditions and with complicated and various forms. The specificity of each site can be well reflected in the representation, and a management system can be established through which effective maintenance management is possible through the prompt identification of the communication relationships between all components.

The SGRS was applied to a WSN employed in an actual large-scale irregular structure in which three types of sensors (75 vibrating wire strain gauges, 10 inclinometers, and three laser displacement sensors) and customized wireless sensor nodes were installed. Despite being a structure with complicated and varied configurations, the communication relationships and respective location information of the system components were concisely expressed in a small space with an emphasis on the flow of measured data. Furthermore, the application results demonstrated that the prompt identification of sensing units and effective management of distributed sensor networks can be realized from the SGRS, thereby confirming its superiority relative to the conventional expression method using a box or tree diagram representing the ID of sensors and data loggers (nodes).

## Figures and Tables

**Table 1.** Notation Method.

**Location**			**Component**		**Master Node**	**Mark 2**	**Example (Mark 1+ Mark 2)**
**Floor**	**Monitoring Unit**	**Mark 1**					
n	xy	$Z_{xy}^{n}$	Sensor (S)	VWSG (V)	δ	$S_{\delta }^{V}\#$	$Z_{xy}^{n}S_{\delta }^{V}1$, $Z_{xy}^{n}S_{\delta }^{V}2$, $Z_{xy}^{n}S_{\delta }^{V}3$, $Z_{xy}^{n}S_{\delta }^{V}4$
				Inclinometer (I)		$S_{\delta }^{I}\#$	$Z_{xy}^{n}S_{\delta }^{I}1$, $Z_{xy}^{n}S_{\delta }^{I}2$, $Z_{xy}^{n}S_{\delta }^{I}3$
				Laser Displacement(D)		$S_{\delta }^{D}\#$	$Z_{xy}^{n}S_{\delta }^{D}1$
			Sensor Node (SN)	VWSG (V)		$SN_{\delta }^{Vi}$	(i: a number of VWSG connected $Z_{xy}^{n}SN_{\delta }^{Vi}$)
				Inclinometer (I)		$SN_{\delta }^{Ij}$	(j: a number of Inclinometer connected $Z_{xy}^{n}SN_{\delta }^{Ij}$)
				Laser Displacement (D)		$SN_{\delta }^{Dk}$	(k: a number of Laser Displacement connected $Z_{xy}^{n}SN_{\delta }^{Dk}$)
			Repeater node (RP)			*RP_δ_*#	$Z_{xy}^{n}RP_{\delta }^{\;}1$, $Z_{xy}^{n}RP_{\delta }^{\;}1$, $Z_{xy}^{n}RP_{\delta }^{\;}1$, …
			Master node (MN)			*MN#*	$Z_{xy}^{n}MN1$, $Z_{xy}^{n}MN2$, $Z_{xy}^{n}MN3$, …

**Table 2.** Notation for the sensor nodes used at D-Building.

**Master Node(11EA)**	**Zone**	**Sensor Node (30 EA)**								

		**From VWSGs**			**From Inclinometers**			**From Laser Displacement Sensors**		
		
		**Mark**	**Floor**	**Number**	**Mark**	**Floor**	**Number**	**Mark**	**Floor**	**Number**
*MN*1*_A_*_1_	A1	$Z_{A1}^{B3}SN_{1}^{V4}$	B3	4						
*MN*2*_A_*_4_	A4	$Z_{A4}^{3}SN_{2}^{V4}$	B3	4	$Z_{A4}^{3}SN_{2}^{I2}$	3	2	$Z_{A4}^{3}SN_{2}^{D1}$	3	1
*MN*3*_B_*_1_	B1	$Z_{B1}^{B3}SN_{3}^{V4}$	B3	4						
	B4	$Z_{B4}^{B3}SN_{3}^{V4}$	B3	4						
*MN*4*_A_*_5_	A5	$Z_{A5}^{4}SN_{4}^{V4}$	4	4	$Z_{A5}^{4}SN_{4}^{I1}$	4	1			
	A8	$Z_{A8}^{R}SN_{4}^{V4}$	R	4						
*MN*5*_B_*_6_	B2	$Z_{B2}^{3}SN_{5}^{V4}$	3	4	$Z_{B2}^{3}SN_{5}^{I2}$	3	2	$Z_{B2}^{3}SN_{5}^{D1}$	3	1
	B5	$Z_{B5}^{3}SN_{5}^{V4}$	3	4	$Z_{B5}^{3}SN_{5}^{I1}$	3	1			
	B6	$Z_{B6}^{3}SN_{5}^{V4}$	3	4	$Z_{B6}^{3}SN_{5}^{I1}$	3	1			
*MN*6*_D_*_2_	D1	$Z_{D1}^{3}SN_{6}^{V3}$	3	3						
	D2	$Z_{D2}^{3}SN_{6}^{V3}$	3	3						
	D3	$Z_{D3}^{3}SN_{6}^{V3}$	3	3	$Z_{D3}^{3}SN_{6}^{I1}$	3	1			
*MN*7*_C_*_2_	C2	$Z_{C2}^{3}SN_{7}^{V3}$	3	3						
	C3	$Z_{C3}^{3}SN_{7}^{V3}$	3	3	$Z_{C3}^{3}SN_{7}^{I1}$	3	1			
*MN*8*_C_*_1_	C1	$Z_{C1}^{3}SN_{8}^{V3}$	3	3	$Z_{C1}^{3}SN_{8}^{I1}$	3	1			
*MN*9*_A_*_3_	A6	$Z_{A6}^{3}SN_{9}^{V2}$	3	2	$Z_{A6}^{3}SN_{9}^{I1}$	3	1			
	A3	$Z_{A3}^{4}SN_{9}^{V4}$	4	4						
	A7	$Z_{A7}^{R}SN_{9}^{V4}$	R	4						
	B3	$Z_{B3}^{R}SN_{9}^{V4}$	R	4						
*MN*10*_A_*_2_	A2	$Z_{A2}^{3}SN_{10}^{V4}$	3	4	$Z_{A2}^{3}SN_{10}^{I2}$	3	2	$Z_{A2}^{3}SN_{10}^{DI}$	3	1
*MN*11*_E_*_3_	E1	$Z_{E1}^{3}SN_{11}^{V3}$	3	3						
	E2	$Z_{E2}^{3}SN_{11}^{V3}$	3	3						
	E3	$Z_{E3}^{3}SN_{11}^{V3}$	3	3						
Total number (EA)		75			10			3		

## References

[1] Maaskant R., Alavie T., Measures R.M., Tadros G., Rizkalla S.H., Guha-Thakurta A. (1997). Fiber-optic bragg grating sensors for bridge monitoring. Cem. Concr. Comp..

[2] Park H.S., Jung S.M., Lee H.M., Kwon Y.H., Seo J.H. (2007). Analytical models for assessment of the safety of multi-span steel beams based on average strains from long gage optic sensors. Sens. Actuators A Phys..

[3] Lee H.M., Park H.S. (2013). Measurement of maximum strain of steel beam structures based on average strains from vibrating wire strain gages. Exp. Technol..

[4] Doebling S.W., Farrar C.R., Prime M.B., Shevitz D.W. (1996). Damage Identification and Health Monitoring of Structural and Mechanical Systems from Change in Their Vibration Characteristics: A Literature Review.

[5] Salawu O.S. (1997). Detection of structural damage through changes in frequency: A review. Eng. Struct..

[6] Park H.S., Lee H.M., Adeli H., Lee I. (2007). A new approach for health monitoring of structures: Terrestrial laser scanning. Comput. Aided Civil Infrastr. Eng..

[7] Nakamura S. (2000). GPS measurement of wind-induced suspension bridge girder displacements. J. Struct. Eng..

[8] Ni Y.Q., Li B., Lam K.H., Zhu D., Wang Y., Lynch J.P., Law K.H. (2010). In-construction vibration monitoring of a super-tall structure using a long-range wireless sensing system. Smart Struct. Syst..

[9] Wu Z.F., Gao F. (2011). Application and research of steel structure construction monitoring of costa rica state stadium canopy with measurement robot. Energy Procedia.

[10] Park H.S., Sohn H.G., Kim I.S., Park J.H. (2007). Application of GPS to monitoring of wind-induced responses of high-rise buildings. Struct. Des. Tall Spec. Build..

[11] Celebi M., Eeri M., Sanli A. (2002). GPS in pioneering dynamic monitoring of long-period structures. Earthq. Spcetra.

[12] Balendra T., Anwar M.P., Tey K.L. (2005). Direct measurement of wind-induced displacement in tall building models using laser positioning technique. J. Wind Eng. Ind. Aerodyn..

[13] Ou J., Li H. (2010). Structural health monitoring in mainland china: Review and future trends. Struct. Health Monit..

[14] Lynch J.P., Loh K.J. (2006). A summary review of wireless sensors and sensor networks for structural health monitoring. Shock Vib. Digest.

[15] Xu N., Rangwala S., Chintalapudi K.K., Ganesan D., Broad A., Govindan R., Estrin D. A Wireless Sensor Network for Structural Monitoring.

[16] Lee H.M., Kim J.M., Sho K., Park H.S. (2010). A wireless vibrating wire sensor node for continuous structural health monitoring. Smart Mater. Struct..

[17] Hu X.Y., Wang B.W., Ji H. (2013). A wireless sensor network-based structural health monitoring system for highway bridges. Comput.-Aided Civil Infrastruct. Eng..

[18] Tubaishat M., Madria S. (2003). Sensor networks: An overview. Potentials IEEE.

[19] Merrett G.V., Harris N.R., Al-Hashimi B.M., White N.M. (2008). Energy managed reporting for wireless sensor networks. Sens. Actuators A Phys..

[20] Zhong C., Worboys M. Generating Contours in a Sensor Network using Isovector Aggregation.

[21] Suryadevara N.K., Mukhopadhyay S.C. (2012). Wireless sensor network based home monitoring system for wellness determination of elderly. IEEE Sens. J..

[22] Jabbari A., Jedermann R., Muthuraman R., Lang W. (2009). Application of neurocomputing for data approximation and classification in wireless sensor networks. Sensors.

[23] Chen J., Salim M.B., Matsumoto M. (2010). Modeling the energy performance of event-driven wireless sensor network by using static sink and mobile sink. Sensors.

[24] Ha S.W., Lee Y.K., Vu T.H.N., Jung Y.J., Ryu K.H. (2012). An environmental monitoring system for managing spatiotemporal sensor data over sensor networks. Sensors.

[25] Ni Y.Q., Xia Y., Liao W.Y., Ko J.M. (2009). Technology innovation in developing the structural health monitoring system for Guangzhou New TV Tower. Struct. Control Health Monit..

[26] Xia Y., Ni Y.Q., Zhang P., Liao W.Y., Ko J.M. (2011). Stress development of a supertall structure during construction: Field monitoring and numerical analysis. Comput.-Aided Civil Infrastruct. Eng..

[27] Su J.Z., Xia Y., Chen L., Zhao X., Zhang Q.L., Xu Y.L., Chen A.R. (2013). Long-term structural performance monitoring system for the Shanghai Tower. J. Civil Struct. Health Monit..

[28] Kurata N., Suzuki M., Saruwatari S., Morikawa H. Actual Application of Ubiquitous Structural Monitoring System using Wireless Sensor Networks.

